# Supplementary material for: Systematic review and meta-analysis of birth outcomes in women with polycystic ovary syndrome
Source: Nat Commun. 2024 Jul 4;15:5592. doi: 10.1038/s41467-024-49752-6 (PMC11224419; doi:10.1038/s41467-024-49752-6)
Supplement: Supplementary file 1 — Supplementary Information [file 41467_2024_49752_MOESM1_ESM.pdf]

Systematic Review and Meta-Analysis of Birth Outcomes in Women with Polycystic Ovary Syndrome

Supplementary Table 1. Characteristics of included studies

Study	Country	Design	Quality	PCOS	Controls	Matched characteristics	Outcomes
Diamant et al (1982)	Israel	Prospective	Poor	N: 70<br>Age*: 31<br>BMI†: NR	N: 2071<br>Age: 30<br>BMI: NR	-	BW
Wortsman et al (1991)	USA	Retrospective cohort	Poor	N: 53<br>Age: 29<br>BMI: NR	N: 2036<br>Age: NR<br>BMI: NR	-	BW<br>Macrosomia
Lesser et al (1997)	USA	Retrospective cohort	Poor	N: 24<br>Age: 30<br>BMI: 28	N: 44<br>Age: 32<br>BMI: 23	-	PTB
Urman et al (1997)	Turkey	Retrospective cohort	Moderate	N: 47<br>Age: 28<br>BMI: 25	N: 100<br>Age: 28<br>BMI: 23	-	PTB<br>LBW<br>Macrosomia
Fridstrom et al (1999)	Sweden	Retrospective cohort	Poor	N: 9<br>Age: 32<br>BMI: 25	N: 10<br>Age: 33<br>BMI: 23	Age	PTB<br>BW
Vollenhoven et al (2000)	Australia	Retrospective cohort	Moderate	N: 60<br>Age: NR<br>BMI: 27	N: 60<br>Age: NR<br>BMI: 27	Age<br>BMI<br>Ethnicity	BW
Mikola et al (2001)	Finland	Retrospective cohort	Poor	N: 99<br>Age: 30<br>BMI: 26	N: 737<br>Age: 29<br>BMI: 23	-	PTB<br>BW<br>Macrosomia
Bjercke et al (2002)	Norway	Retrospective cohort	Poor	N: 52<br>Age: 31<br>BMI: 26	N: 355<br>Age: 33<br>BMI: 22	-	PTB<br>BW
Haakova et al (2003)	Czech Republic	Retrospective cohort	High	N: 66<br>Age: 29<br>BMI: 24	N: 66<br>Age: 30<br>BMI: 23	Age<br>Weight	PTB<br>BW
Turhan et al (2003)	Turkey	Retrospective cohort	Poor	N: 38<br>Age: 28<br>BMI: 32	N: 136<br>Age: 27<br>BMI: 24	-	PTB<br>IUGR<br>BW<br>Macrosomia
Glueck et al (2004)	USA	Prospective	Poor	N: 119<br>Age: 31<br>BMI: 34	N: 251<br>Age: 29<br>BMI: 26	-	PTB
Glueck et al (2004)	USA	Prospective	Poor	N: 95<br>Age: 33<br>BMI: 34	N: 251<br>Age: 29<br>BMI: 26	-	PTB<br>BW<br>Macrosomia
Sir-Petermann et al (2005)	Chile	Prospective	Poor	N: 47<br>Age: 25<br>BMI: 28	N: 180<br>Age: 26<br>BMI: 26	Age<br>BMI<br>SES	PTB<br>BW<br>SGA<br>LGA
Al-Ojaimi et al (2006)	Bahrain	Prospective	Poor	N: 134<br>Age: 29<br>BMI: 31	N: 479<br>Age: 28<br>BMI: 29	-	PTB<br>BW<br>LBW<br>Macrosomia
Dokras et al (2006)	USA	Retrospective cohort	High	N: 46<br>Age: NR<br>BMI: NR	N: 108<br>Age: NR<br>BMI: NR	Weight	PTB
Kovo et al (2006)	Israel	Retrospective cohort	Moderate	N: 33<br>Age: 30<br>BMI: 28	N: 66<br>Age: 31<br>BMI: 25	Age	PTB<br>BW
Hu et al (2007)	UK	Prospective	High	N: 22<br>Age: 32<br>BMI: 24	N: 22<br>Age: 32<br>BMI: 24	Age<br>BMI<br>Ethnicity<br>Parity	BW
Bolton et al (2009)	Ireland	Retrospective cohort	Poor	N: 66<br>Age: 32	N: 66<br>Age: 32	Age<br>Parity	PTB<br>BW

				BMI: NR	BMI: NR		SGA<br>LGA
Maliqueo et al (2009)	Chile	Prospective	Moderate	N: 30<br>Age: 27<br>BMI: 29	N: 34<br>Age: 27<br>BMI: 24	SES	BW<br>SGA<br>LGA
Anderson et al (2010)	USA	Prospective	Poor	N: 39<br>Age: 30<br>BMI: 31	N: 31<br>Age: 32<br>BMI: 25	-	BW<br>LGA<br>SGA
Falbo et al (2010)	Italy	Prospective	High	N: 45<br>Age: 28<br>BMI: 25	N: 42<br>Age: 28<br>BMI: 25	Age<br>BMI	BW
Li et al (2010)	China	Retrospective cohort	Poor	N: 34<br>Age: 32<br>BMI: NR	N: 70<br>Age: 32<br>BMI: NR	-	PTB<br>BW<br>SGA<br>Macrosomia<br>LGA
Palomba et al (2010)	Italy	Prospective	Poor	N: 93<br>Age: NR<br>BMI: NR	N: 69<br>Age: NR<br>BMI: NR	-	PTB<br>IUGR<br>SGA<br>LGA
Palomba et al (2010)	Italy	Prospective	High	N: 70<br>Age: NR<br>BMI: NR	N: 69<br>Age: NR<br>BMI: NR	Age<br>BMI	PTB<br>IUGR<br>SGA<br>LGA
De Leo et al (2011)	Italy	Prospective	Poor	N: 98<br>Age: 32<br>BMI: 28	N: 110<br>Age: 33<br>BMI: 27	-	PTB<br>BW
Dmitrovic et al (2011)	USA	Retrospective cohort	Poor	N: 17<br>Age: 29<br>BMI: 32	N: 17<br>Age: 31<br>BMI: 26	-	PTB<br>BW<br>SGA<br>LGA
Nouh et al (2011)	Egypt	Prospective	High	N: 40<br>Age: NR<br>BMI: NR	N: 40<br>Age: NR<br>BMI: NR	Age<br>BMI	PTB<br>SGA<br>LGA
Mehrabian et al (2012)	Iran	Retrospective cohort	High	N: 40<br>Age: 27<br>BMI: 26	N: 40<br>Age: 28<br>BMI: 26	Age<br>BMI<br>SES	BW
Palomba et al (2012)	Italy	Prospective	High	N: 42<br>Age: 28<br>BMI: 28	N: 84<br>Age: 28<br>BMI: 27	Age<br>BMI	PTB<br>BW<br>SGA<br>Macrosomia<br>LGA
Reyes-Munoz et al (2012)	Mexico	Retrospective cohort	Moderate	N: 52<br>Age: 29<br>BMI: 28	N: 52<br>Age: 29<br>BMI: 28	Age<br>BMI<br>Parity	PTB<br>BW<br>SGA<br>LGA
Yamamoto et al (2012)	USA	Retrospective cohort	Poor	N:908<br>Age:<br>BMI:	N:992<br>Age:<br>BMI:	-	PTB
Boutzios et al (2013)	Greece	Prospective	Poor	N: 41<br>Age: 31<br>BMI: 25	N: 110<br>Age: 32<br>BMI: 24	-	BW<br>SGA<br>LGA
Wang et al (2013)	China	Prospective	High	N: 220<br>Age: 31<br>BMI: 23	N: 652<br>Age: 29<br>BMI: 20	-	PTB<br>IUGR<br>Macrosomia
Elkholi et al (2014)	Egypt	Prospective	Moderate	N: 200<br>Age: 23<br>BMI: 32	N: 200<br>Age: 23<br>BMI: 32	Age<br>BMI<br>SES	PTB<br>BW<br>IUGR<br>Macrosomia
Foroozanfard et al (2014)	Iran	Retrospective cohort	Moderate	N: 130<br>Age: 29<br>BMI: 28	N: 131<br>Age: 29<br>BMI: 28	-	PTB<br>BW<br>Macrosomia
Naver et al (2014)	Denmark	Prospective	Poor	N: 459<br>Age: 32<br>BMI: 23	N: 5409<br>Age: 31<br>BMI: 23	-	PTB<br>BW<br>SGA<br>LGA

Palomba et al (2014)	Italy	Prospective	High	N: 150<br>Age: 28<br>BMI: 27	N: 150<br>Age: 27<br>BMI: 27	Age<br>BMI	BW<br>LGA
Palomba et al (2014)	Italy	Prospective	High	N: 150<br>Age: 28<br>BMI: NR	N: 150<br>Age: 28<br>BMI: NR	Age<br>BMI	PTB<br>IUGR<br>SGA
Kollmann et al (2015)	Austria	Retrospective cohort	Poor	N: 177<br>Age: NR<br>BMI: NR	N: 708<br>Age: NR<br>BMI: NR	-	PTB<br>SGA<br>LGA
Koster et al (2015)	Netherlands	Prospective	Poor	N: 73<br>Age: 31<br>BMI: NR	N: 209<br>Age: 32<br>BMI: NR	-	BW<br>SGA<br>LGA
Mumm et al (2015)	Denmark	Prospective	Poor	N: 157<br>Age: NR<br>BMI: NR	N: 1037<br>Age: NR<br>BMI: NR	-	PTB<br>SGA<br>LGA
Sawada et al (2015)	Japan	Retrospective cohort	High	N: 49<br>Age: 32<br>BMI: 24	N: 49<br>Age: 32<br>BMI: 24	Age<br>BMI<br>Parity	PTB<br>BW<br>IUGR
Wan et al (2015)	China	Retrospective cohort	Moderate	N: 24<br>Age: 31<br>BMI: 23	N: 224<br>Age: 33<br>BMI: 21	Age	PTB<br>BW
Aktun et al (2016)	Turkey	Prospective	Poor	N: 150<br>Age: 29<br>BMI: 23	N: 160<br>Age: 31<br>BMI: 21	-	PTB<br>Macrosomia
Sterling et al (2016)	Canada	Retrospective cohort	Poor	N: 71<br>Age: NR<br>BMI: 25	N: 323<br>Age: NR<br>BMI: 24	-	PTB<br>LBW<br>SGA<br>Macrosomia<br>LGA
Xiao et al (2016)	China	Retrospective cohort	Poor	N: 352<br>Age: 30<br>BMI: NR	N: 2037<br>Age: 29<br>BMI: NR	-	PTB<br>BW<br>LBW<br>SGA<br>Macrosomia<br>LGA
deWilde et al (2017)	Netherlands	Prospective	Poor	N: 188<br>Age: NR<br>BMI: NR	N: 2889<br>Age: NR<br>BMI: NR	-	PTB<br>SGA<br>LGA
Jonsdottir et al (2017)	Denmark	Retrospective cohort	Moderate	N: 72<br>Age: 33<br>BMI: 22	N: 288<br>Age: 32<br>BMI: 23	-	PTB<br>BW<br>LBW<br>SGA
Kent et al (2018)	USA	Retrospective cohort	Moderate	N: 164<br>Age: 28<br>BMI: 33	N: 154<br>Age: 32<br>BMI: 28	-	SGA<br>LGA
Li et al (2018)	China	Retrospective cohort	Poor	N: 670<br>Age: 31<br>BMI: 24	N: 6000<br>Age: 32<br>BMI: 22	-	PTB<br>LBW<br>Macrosomia
Zheng et al (2019)	China	Retrospective cohort	High	N: 242<br>Age: 30<br>BMI: 24	N: 324<br>Age: 31<br>BMI: 25	-	PTB<br>BW<br>LBW<br>SGA<br>Macrosomia<br>LGA
Benito et al (2020)	Spain	Prospective	Moderate	N: 20<br>Age: 34<br>BMI: 29	N: 30<br>Age: 34<br>BMI: 29	-	PTB<br>BW<br>LBW
Chen et al (2020)	China	Prospective	Poor	N: 35<br>Age: NR<br>BMI: NR	N: 29<br>Age: NR<br>BMI: NR	-	PTB<br>BW
Foroozanfard et al (2020)	Iran	Prospective	Poor	N: 41<br>Age: 25<br>BMI: 26	N: 47<br>Age: 26<br>BMI: 24	-	PTB<br>BW<br>IUGR<br>LGA
Liu et al (2020)	China	Retrospective cohort	Poor	N: 472<br>Age: NR	N: 4190<br>Age: NR	-	PTB

				BMI: NR	BMI: NR		
Tobiasz et al (2020)	USA	Prospective	Poor	N: 17<br>Age: NR<br>BMI: NR	N: 11<br>Age: NR<br>BMI: NR	-	PTB<br>IUGR
Abdulkhalikova et al (2021)	Slovenia	Retrospective cohort	Poor	N: 73<br>Age: 33<br>BMI: NR	N: 196<br>Age: 34<br>BMI: NR	-	PTB<br>BW<br>LBW<br>SGA<br>Macrosomia
Cai et al (2021)	China	Retrospective cohort	Moderate	N: 2357<br>Age: 29<br>BMI: 24	N: 19463<br>Age: 31<br>BMI: 22	-	PTB<br>BW<br>LBW<br>Macrosomia
Diboun et al (2021)	Qatar	Prospective	Moderate	N:16<br>Age:34±5<br>BMI:34±7	N:52<br>Age:31±6<br>BMI:32±7	Age<br>BMI<br>Weight	BW
Gongadashetti et al (2021)	India	Prospective	Poor	N: 12<br>Age: NR<br>BMI: NR	N: 14<br>Age: NR<br>BMI: NR	-	PTB
Hu et al (2021)	China	Retrospective cohort	Poor	N: 557<br>Age: 30<br>BMI: 23	N: 3526<br>Age: 31<br>BMI: 22	-	PTB<br>BW<br>LBW<br>Macrosomia
Jiang et al (2021)	China	Retrospective cohort	Poor	N: 30<br>Age: 31<br>BMI: 25	N: 31<br>Age: 32<br>BMI: 23	-	PTB<br>LBW<br>Macrosomia
Kaing et al (2021)	USA	Retrospective cohort	Poor	N:118<br>Age:29±4<br>BMI:32±9	N:146<br>Age:32±4<br>BMI:27±6	-	PTB<br>BW
Kollmann et al (2021)	Austria	Retrospective cohort	Moderate	N: 79<br>Age: 31<br>BMI: 30	N: 354<br>Age: 30<br>BMI: 29	-	IUGR<br>SGA<br>LGA
Lin et al (2021)	China	Retrospective cohort	Poor	N:1167<br>Age:<br>BMI:	N:9995<br>Age:<br>BMI:	-	PTB<br>BW<br>LBW<br>SGA<br>Macrosomia<br>LGA
Wang et al (2021)	China	Retrospective cohort	Moderate	N: 29<br>Age: NR<br>BMI: 22	N: 116<br>Age: NR<br>BMI: 21	-	BW
Wu et al (2021)	China	Retrospective cohort	High	N: 23<br>Age: 30<br>BMI: 23	N: 11<br>Age: 30<br>BMI: 22	Age<br>BMI<br>Parity	PTB
Zhu et al (2021)	China	Retrospective cohort	Poor	N: 111<br>Age: NR<br>BMI: NR	N: 237<br>Age: NR<br>BMI: NR	-	PTB<br>LBW
Tu et al (2022)	China	Retrospective cohort	Poor	N: 27<br>Age: 29<br>BMI: 22	N: 22<br>Age: 30<br>BMI: 21	-	BW
Liu et al (2022)	China	Retrospective cohort	Poor	N: 1357<br>Age: 31<br>BMI: NR	N: 6940<br>Age: 32<br>BMI: NR	-	PTB<br>IUGR<br>Macrosomia
Ni t al (2022)	China	Retrospective cohort	Poor	N: 1376<br>Age: 31<br>BMI: 24	N: 1376<br>Age: 31<br>BMI: 22	-	PTB<br>LBW<br>Macrosomia
Stokkeland et al (2022)	Norway<br>Sweden<br>Iceland	Retrospective cohort	Poor	N:358<br>Age:30±4<br>BMI:	N:258<br>Age:29±4<br>BMI:	-	BW
Wang et al (2022)	China	Retrospective cohort	Poor	N: 346<br>Age: 29<br>BMI: 25	N: 453<br>Age: 29<br>BMI: 23	-	PTB

BMI: body mass index; PTB: preterm birth; BW: birthweight; IUGR: intrauterine growth restriction; LBW: low birth weight; SGA: small for gestational age; LGA: large for gestational age NR: not reported; PCOS: polycystic ovary syndrome; SES: socioeconomic status

*Age is reported in years

†BMI is reported in kg/m^2

Supplementary Figure 1_a. Forest Plot of association of polycystic ovary syndrome with preterm birth

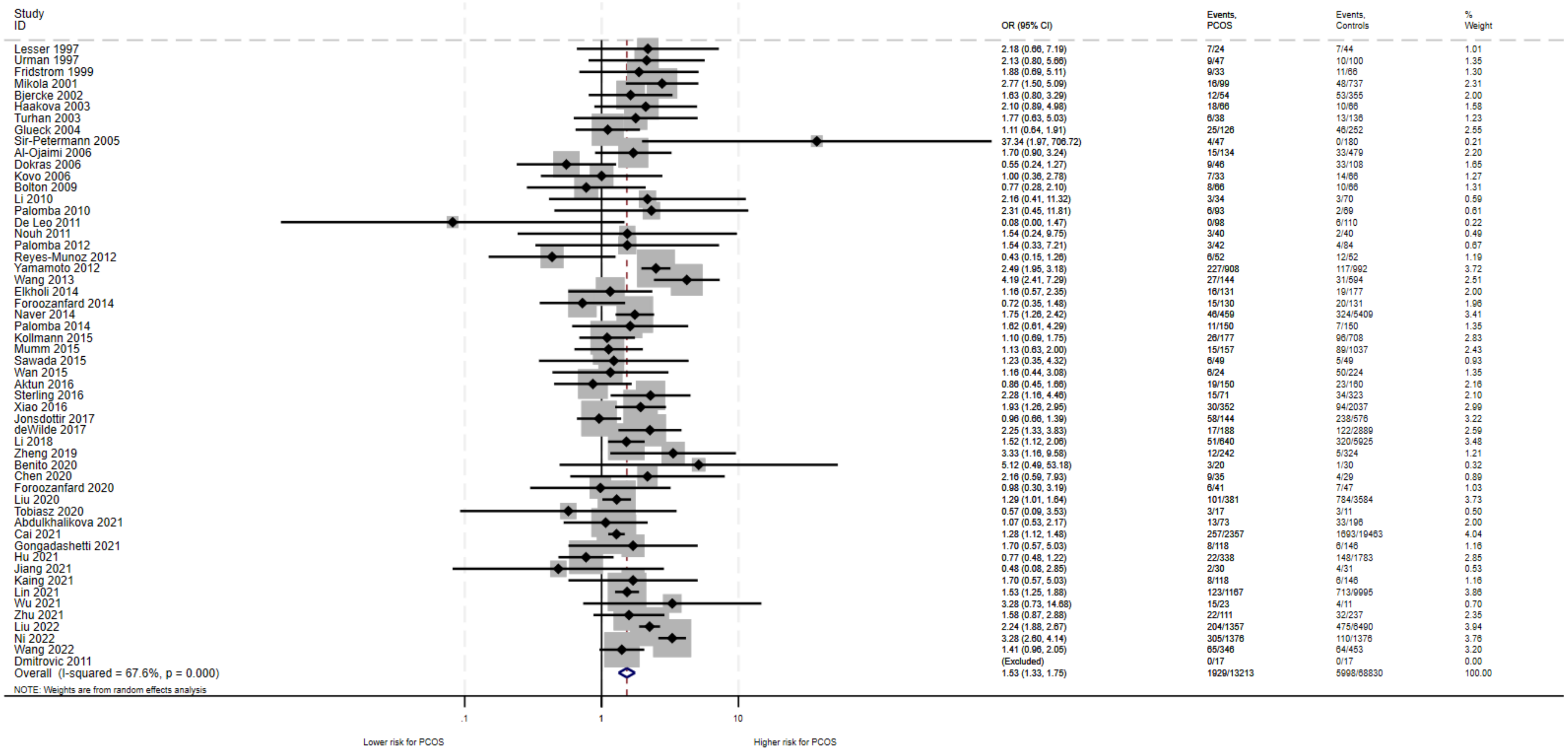

Study ID	OR (95% CI)	Events, PCOS	Events, Controls	% Weight
Lesser 1997	2.18 (0.66, 7.19)	7/24	7/44	1.01
Urman 1997	2.13 (0.80, 5.66)	9/47	10/100	1.35
Fridstrom 1999	1.88 (0.69, 5.11)	9/33	11/66	1.30
Mikola 2001	2.77 (1.50, 5.09)	16/99	48/737	2.31
Bjercke 2002	1.63 (0.80, 3.29)	12/54	53/355	2.00
Haakova 2003	2.10 (0.89, 4.98)	18/66	10/66	1.58
Turhan 2003	1.77 (0.63, 5.03)	6/38	13/136	1.23
Glueck 2004	1.11 (0.64, 1.91)	25/126	46/252	2.55
Sir-Petermann 2005	37.34 (1.97, 706.72)	4/47	0/180	0.21
Al-Ojaimi 2006	1.70 (0.90, 3.24)	15/134	33/479	2.20
Dokras 2006	0.55 (0.24, 1.27)	9/46	33/108	1.65
Kovo 2006	1.00 (0.36, 2.78)	7/33	14/66	1.27
Bolton 2009	0.77 (0.28, 2.10)	8/66	10/66	1.31
Li 2010	2.16 (0.41, 11.32)	3/34	3/70	0.59
Palomba 2010	2.31 (0.45, 11.81)	6/93	2/69	0.61
De Leo 2011	0.08 (0.00, 1.47)	0/98	6/110	0.22
Nouh 2011	1.54 (0.24, 9.75)	3/40	2/40	0.49
Palomba 2012	1.54 (0.33, 7.21)	3/42	4/84	0.67
Reyes-Munoz 2012	0.43 (0.15, 1.26)	6/52	12/52	1.19
Yamamoto 2012	2.49 (1.95, 3.18)	227/908	117/992	3.72
Wang 2013	4.19 (2.41, 7.29)	27/144	31/594	2.51
Elkholi 2014	1.16 (0.57, 2.35)	16/131	19/177	2.00
Foroozanfard 2014	0.72 (0.35, 1.48)	15/130	20/131	1.96
Naver 2014	1.75 (1.26, 2.42)	46/459	324/5409	3.41
Palomba 2014	1.62 (0.61, 4.29)	11/150	7/150	1.35
Kollmann 2015	1.10 (0.69, 1.75)	26/177	96/708	2.83
Mumm 2015	1.13 (0.63, 2.00)	15/157	89/1037	2.43
Sawada 2015	1.23 (0.35, 4.32)	6/49	5/49	0.93
Wan 2015	1.16 (0.44, 3.08)	6/24	50/224	1.35
Aktun 2016	0.86 (0.45, 1.66)	19/150	23/160	2.16
Sterling 2016	2.28 (1.16, 4.46)	15/71	34/323	2.10
Xiao 2016	1.93 (1.26, 2.95)	30/352	94/2037	2.99
Jonsdottir 2017	0.96 (0.66, 1.39)	58/144	238/576	3.22
deWilde 2017	2.25 (1.33, 3.83)	17/188	122/2889	2.59
Li 2018	1.52 (1.12, 2.06)	51/640	320/5925	3.48
Zheng 2019	3.33 (1.16, 9.58)	12/242	5/324	1.21
Benito 2020	5.12 (0.49, 53.18)	3/20	1/30	0.32
Chen 2020	2.16 (0.59, 7.93)	9/35	4/29	0.89
Foroozanfard 2020	0.98 (0.30, 3.19)	6/41	7/47	1.03
Liu 2020	1.29 (1.01, 1.64)	101/381	784/3584	3.73
Tobiasz 2020	0.57 (0.09, 3.53)	3/17	3/11	0.50
Abdulkhalikova 2021	1.07 (0.53, 2.17)	13/73	33/196	2.00
Cai 2021	1.28 (1.12, 1.48)	257/2357	1693/19463	4.04
Gongadashetti 2021	1.70 (0.57, 5.03)	8/118	6/146	1.16
Hu 2021	0.77 (0.48, 1.22)	22/338	148/1783	2.85
Jiang 2021	0.48 (0.08, 2.85)	2/30	4/31	0.53
Kaing 2021	1.70 (0.57, 5.03)	8/118	6/146	1.16
Lin 2021	1.53 (1.25, 1.88)	123/1167	713/9995	3.86
Wu 2021	3.28 (0.73, 14.68)	15/23	4/11	0.70
Zhu 2021	1.58 (0.87, 2.88)	22/111	32/237	2.35
Liu 2022	2.24 (1.88, 2.67)	204/1357	475/6490	3.94
Ni 2022	3.28 (2.60, 4.14)	305/1376	110/1376	3.76
Wang 2022	1.41 (0.96, 2.05)	65/346	64/453	3.20
Dmitrovic 2011	(Excluded)	0/17	0/17	0.00
Overall (I-squared = 67.6%, p = 0.000)	1.53 (1.33, 1.75)	1929/13213	5998/68830	100.00

NOTE: Weights are from random effects analysis

Supplementary Figure 1_b Cumulative plot of association of polycystic ovary syndrome with preterm birth

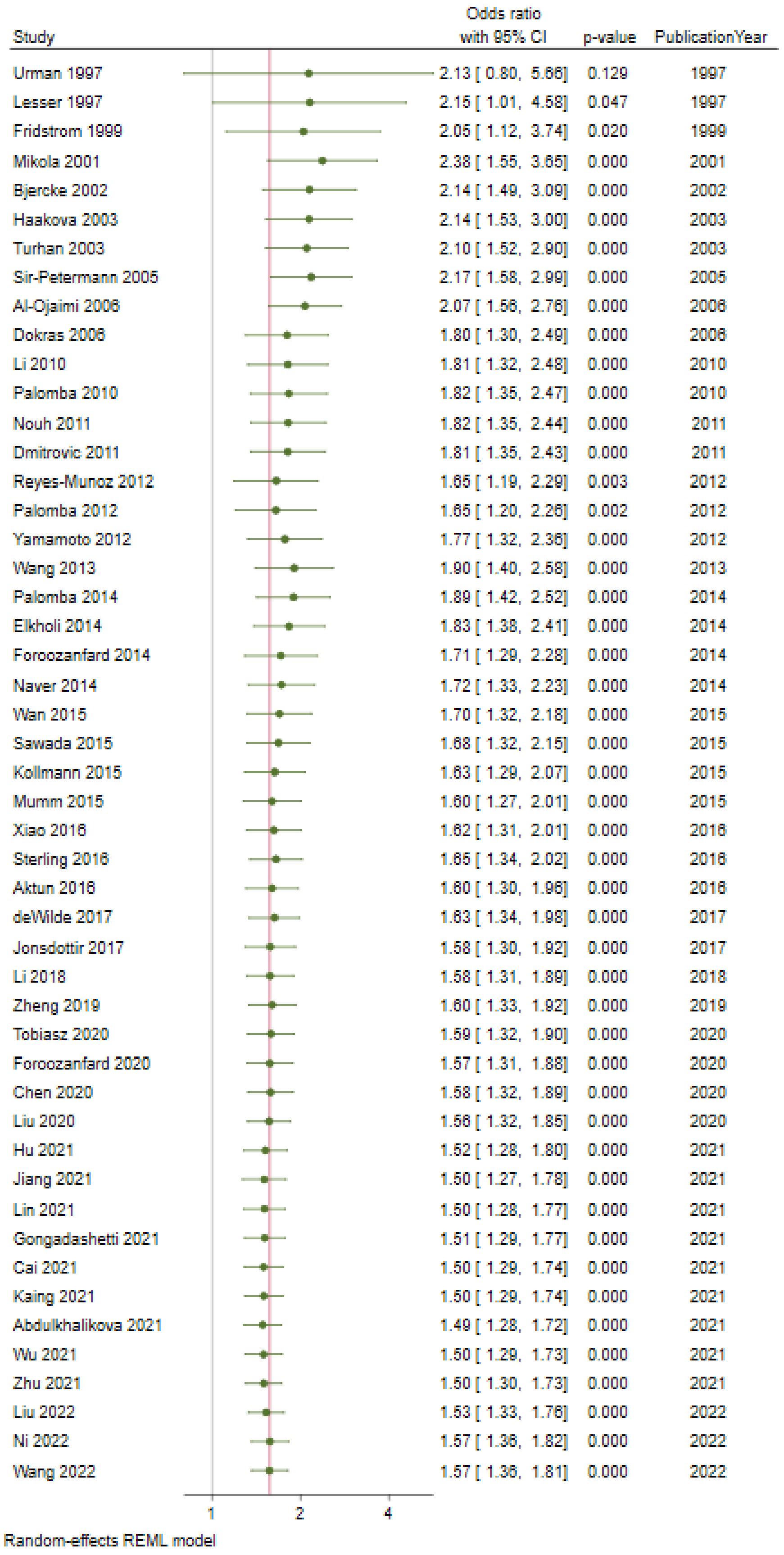

Supplementary Figure 1_c. Funnel plot assessing publication bias in studies on the association of polycystic ovary syndrome with preterm birth

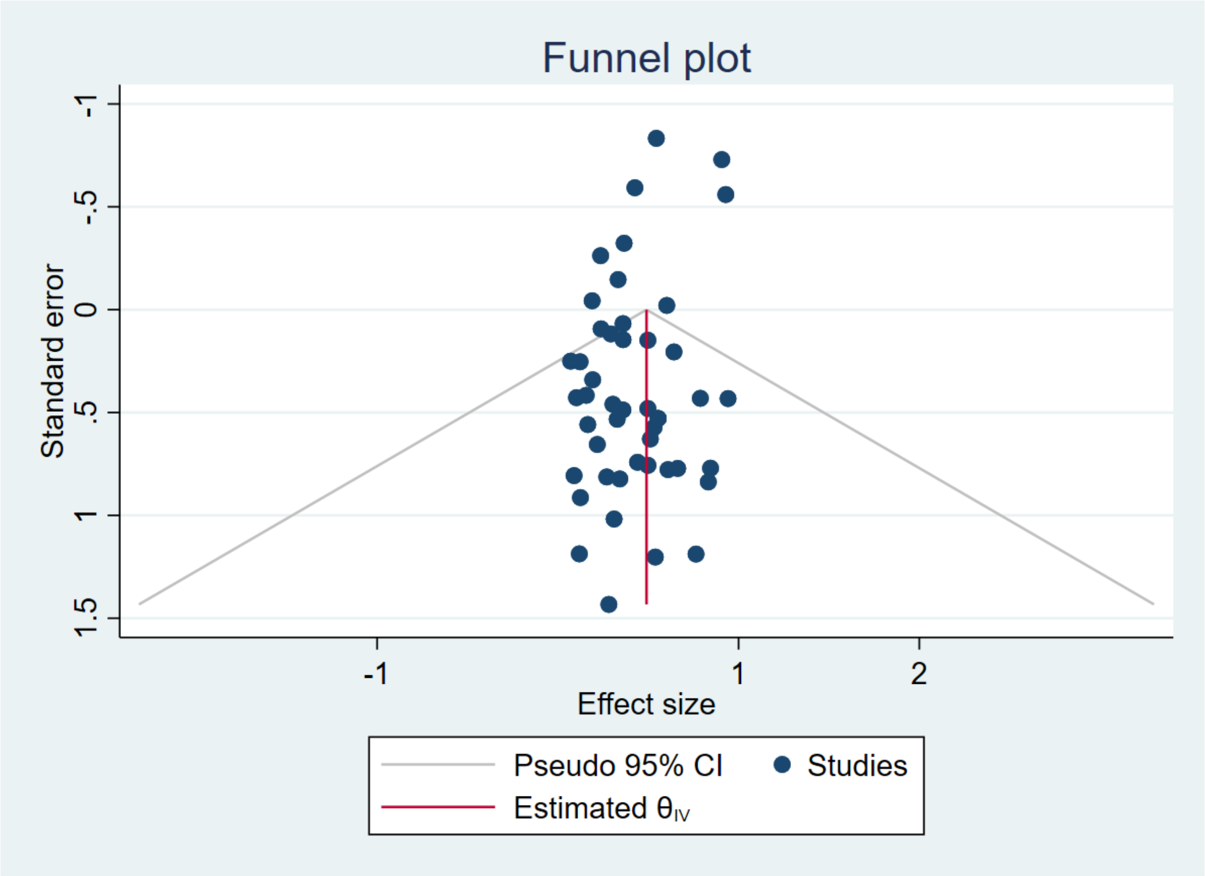

Source data are provided as a Source Data file.

Egger's test for small study effects on the outcome of preterm birth

Number of studies = 48 Root MSE = 1.802

Std_Eff	Coefficient	Std. err.	t	P>|t|	[95% conf. interval]	
slope	0.536161	0.0914259	5.86	0.000	0.3521302	0.7201917
bias	-0.2269485	0.4147166	-0.55	0.587	-1.06173	0.6078327

Test of H0: no small-study effects P = 0.587

Supplementary Figure 2_a. Forest Plot of association of polycystic ovary syndrome with birthweight

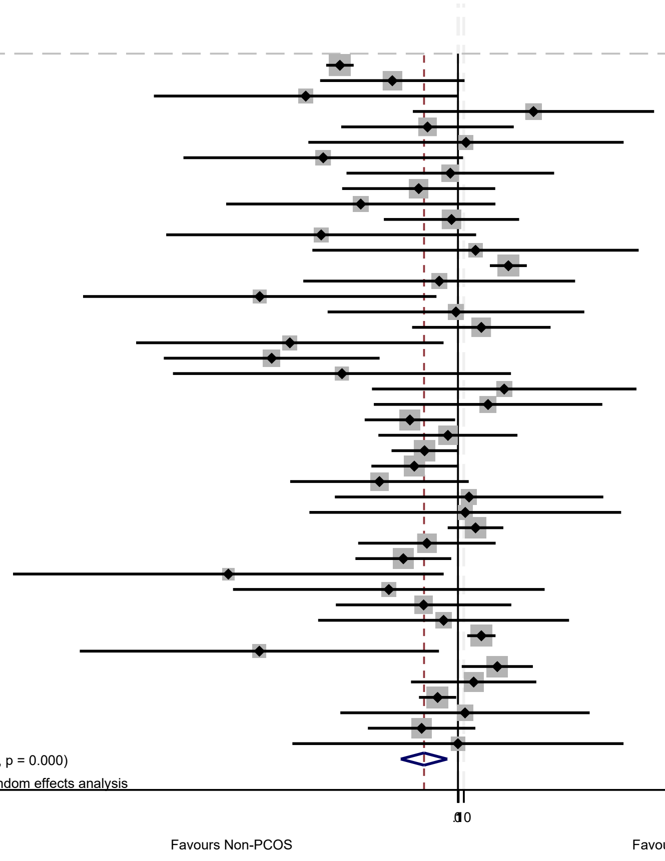

Study ID	WMD (95% CI)	N, mean (SD); PCOS	N, mean (SD); Non-PCOS	% Weight
Diamant 1982	-201.51 (-224.96, -178.07)	70, 3005 (100)	2071, 3207 (20.7)	3.58
Wortsman 1991	-112.00 (-235.30, 11.30)	57, 3353 (468)	2153, 3465 (498)	2.69
Fridstrom 1999	-259.82 (-519.12, -0.52)	33, 3024 (646)	66, 3284 (567)	1.42
Vollenhoven 2000	129.00 (-76.94, 334.94)	60, 3418 (577)	60, 3289 (574)	1.84
Mikola 2001	-52.00 (-199.31, 95.31)	88, 3475 (676)	728, 3527 (576)	2.42
Bjercke 2002	13.77 (-255.42, 282.95)	52, 3289 (945)	355, 3275 (776)	1.36
Haakova 2003	-230.00 (-468.73, 8.73)	61, 3160 (781)	66, 3390 (565)	1.57
Turhan 2003	-13.00 (-190.26, 164.26)	38, 3220 (488)	136, 3233 (510)	2.10
Glueck 2004	-67.00 (-197.71, 63.71)	80, 3414 (486)	206, 3481 (555)	2.60
Sir-Petermann 2005	-165.90 (-395.76, 63.96)	47, 3304 (776)	180, 3469 (415)	1.64
Al-Ojaimi 2006	-11.00 (-126.51, 104.51)	134, 3215 (621)	479, 3226 (534)	2.77
Kovo 2006	-233.50 (-498.10, 31.10)	42, 2585 (710)	84, 2819 (724)	1.39
Bolton 2009	30.00 (-248.61, 308.61)	66, 3330 (711)	66, 3300 (910)	1.30
Maliqueo 2009	86.10 (54.56, 117.64)	30, 3476 (61.8)	34, 3390 (66.9)	3.54
Anderson 2010	-32.00 (-264.07, 200.07)	39, 3539 (597)	31, 3571 (389)	1.62
Falbo 2010	-338.40 (-640.11, -36.69)	45, 3121 (762)	42, 3460 (673)	1.17
Li 2010	-3.40 (-222.54, 215.74)	34, 3422 (570)	70, 3425 (455)	1.72
De Leo 2011	40.00 (-78.22, 158.22)	93, 3150 (350)	76, 3110 (420)	2.74
Dmitrovic 2011	-287.00 (-549.46, -24.54)	17, 3346 (408)	17, 3633 (372)	1.40
Mehrabian 2012	-318.00 (-502.21, -133.79)	40, 2905 (416)	40, 3223 (425)	2.04
Palomba 2012	-198.00 (-486.56, 90.56)	42, 3209 (840)	84, 3407 (640)	1.24
Reyes-Munoz 2012	79.00 (-146.83, 304.83)	52, 3055 (552)	52, 2976 (621)	1.67
Boutzios 2013	51.44 (-143.60, 246.48)	41, 3136 (538)	110, 3085 (559)	1.93
Elkholi 2014	-82.00 (-159.20, -4.81)	131, 3177 (344)	177, 3259 (338)	3.19
Foroozanfard 2014	-17.14 (-135.84, 101.56)	130, 3404 (504)	131, 3421 (474)	2.74
Naver 2014	-57.00 (-113.39, -0.61)	459, 3448 (595)	5409, 3505 (552)	3.38
Palomba 2014	-74.50 (-147.84, -1.16)	150, 3105 (346)	150, 3180 (300)	3.23
Koster 2015	-134.00 (-286.46, 18.46)	73, 3388 (589)	209, 3522 (521)	2.36
Sawada 2015	19.00 (-210.18, 248.18)	49, 2844 (637)	49, 2825 (514)	1.64
Wan 2015	12.48 (-253.66, 278.62)	24, 2722 (628)	224, 2710 (670)	1.38
Xiao 2016	30.00 (-17.48, 77.48)	352, 3253 (422)	2037, 3223 (406)	3.45
Jonsdottir 2017	-53.00 (-170.37, 64.37)	144, 2463 (672)	576, 2516 (509)	2.75
Zheng 2019	-93.20 (-175.02, -11.38)	242, 3391 (546)	324, 3484 (407)	3.14
Benito 2020	-392.00 (-759.64, -24.36)	17, 2763 (618)	27, 3155 (586)	0.88
Chen 2020	-118.00 (-384.03, 148.03)	35, 2979 (434)	29, 3097 (615)	1.38
Foroozanfard 2020	-58.63 (-208.43, 91.17)	43, 3066 (490)	47, 3124 (110)	2.39
Abdulkhalikova 2021	-24.45 (-238.66, 189.77)	73, 3048 (822)	196, 3072 (726)	1.76
Cai 2021	40.00 (15.86, 64.14)	2357, 3340 (570)	19463, 3300 (520)	3.58
Diboun 2021	-339.00 (-645.61, -32.39)	16, 2794 (553)	52, 3133 (529)	1.15
Hu 2021	67.20 (6.45, 127.95)	338, 3418 (522)	1783, 3351 (523)	3.34
Kaing 2021	26.85 (-80.13, 133.83)	118, 3393 (426)	146, 3366 (459)	2.87
Lin 2021	-34.72 (-66.48, -2.96)	1167, 3312 (527)	9995, 3347 (492)	3.54
Wang 2021	12.07 (-200.84, 224.98)	29, 3354 (555)	116, 3342 (372)	1.77
Stokkeland 2022	-62.00 (-153.78, 29.78)	357, 3495 (675)	241, 3557 (470)	3.04
Tu 2022	0.00 (-282.72, 282.72)	24, 3240 (480)	15, 3240 (410)	1.28
Overall (I-squared = 87.6%, p = 0.000)	-57.87 (-97.57, -18.17)	8997	58106	100.00

NOTE: Weights are from random effects analysis

Supplementary Figure 2_b. Cumulative plot of association of polycystic ovary syndrome with birthweight

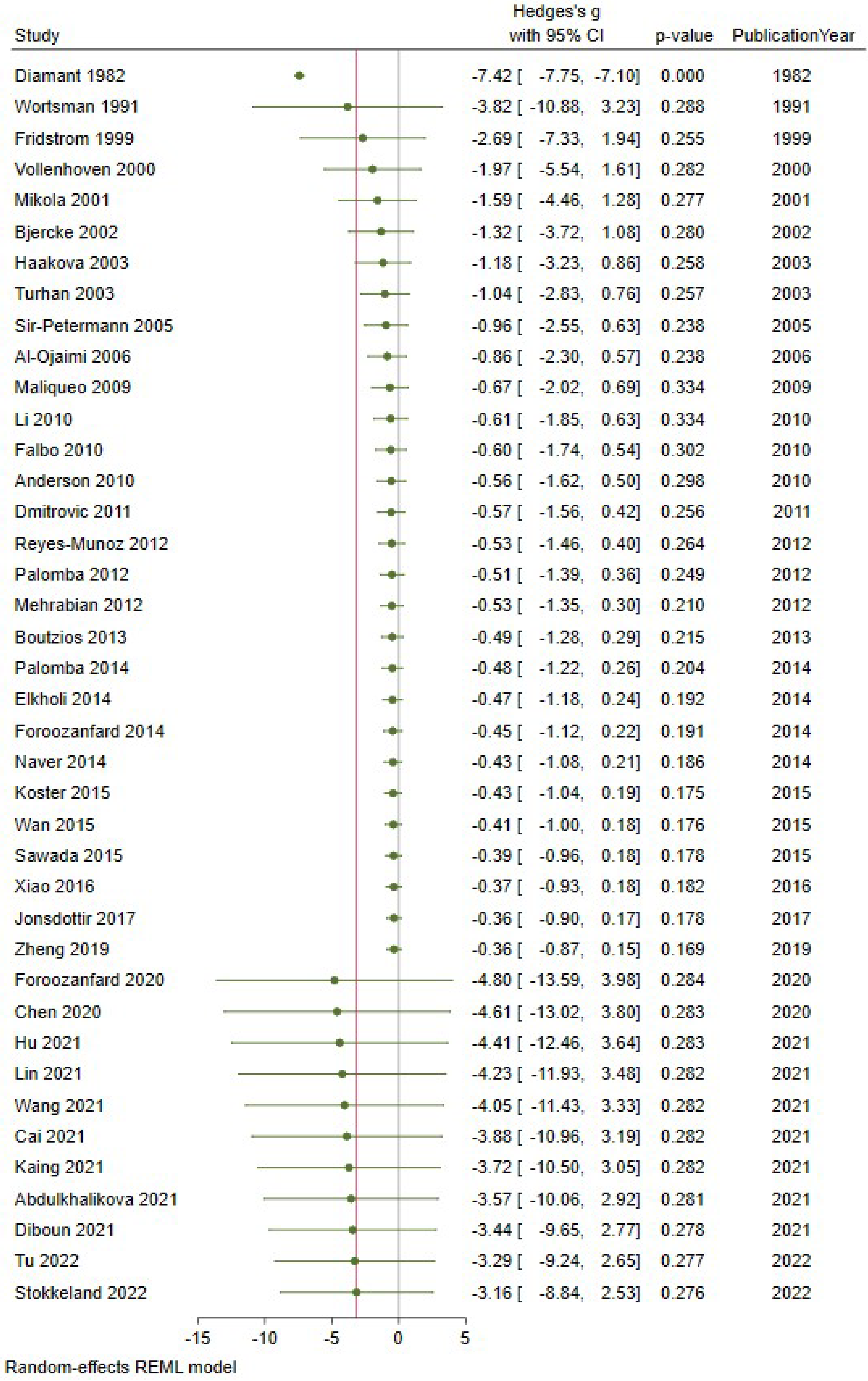

Supplementary Figure 2_c. Funnel plot assessing publication bias in studies on the association of polycystic ovary syndrome with birthweight

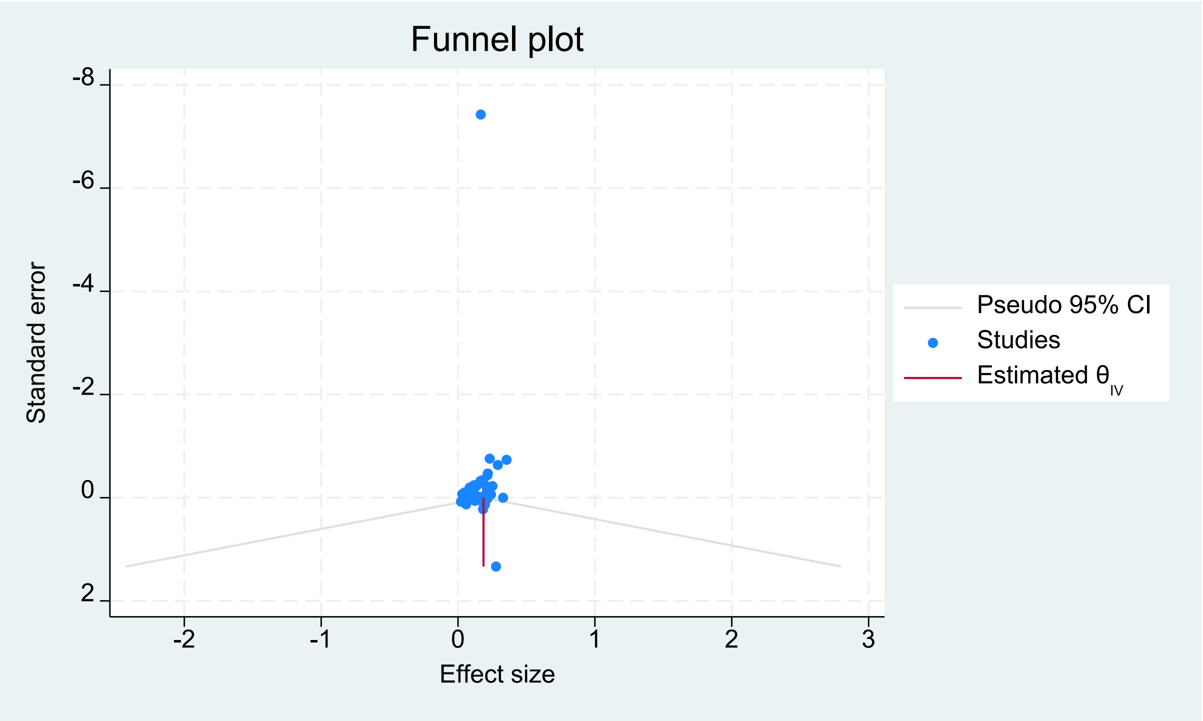

Source data are provided as a Source Data file.

Egger's test for small study effects on the outcome of birthweight

Number of studies = 40 Root MSE = 7.212

Std_Eff	Coefficient	Std. err.	t	P>|t|	[95% conf. interval]	
slope	0.0924857	0.140674	0.66	0.515	-0.1922939	0.3772654
bias	-2.481129	1.682071	-1.48	0.148	-5.886304	0.9240463

Test of H0: no small-study effects P = 0.148

Supplementary Figure 3_a. Forest Plot of association of polycystic ovary syndrome with fetal growth restriction

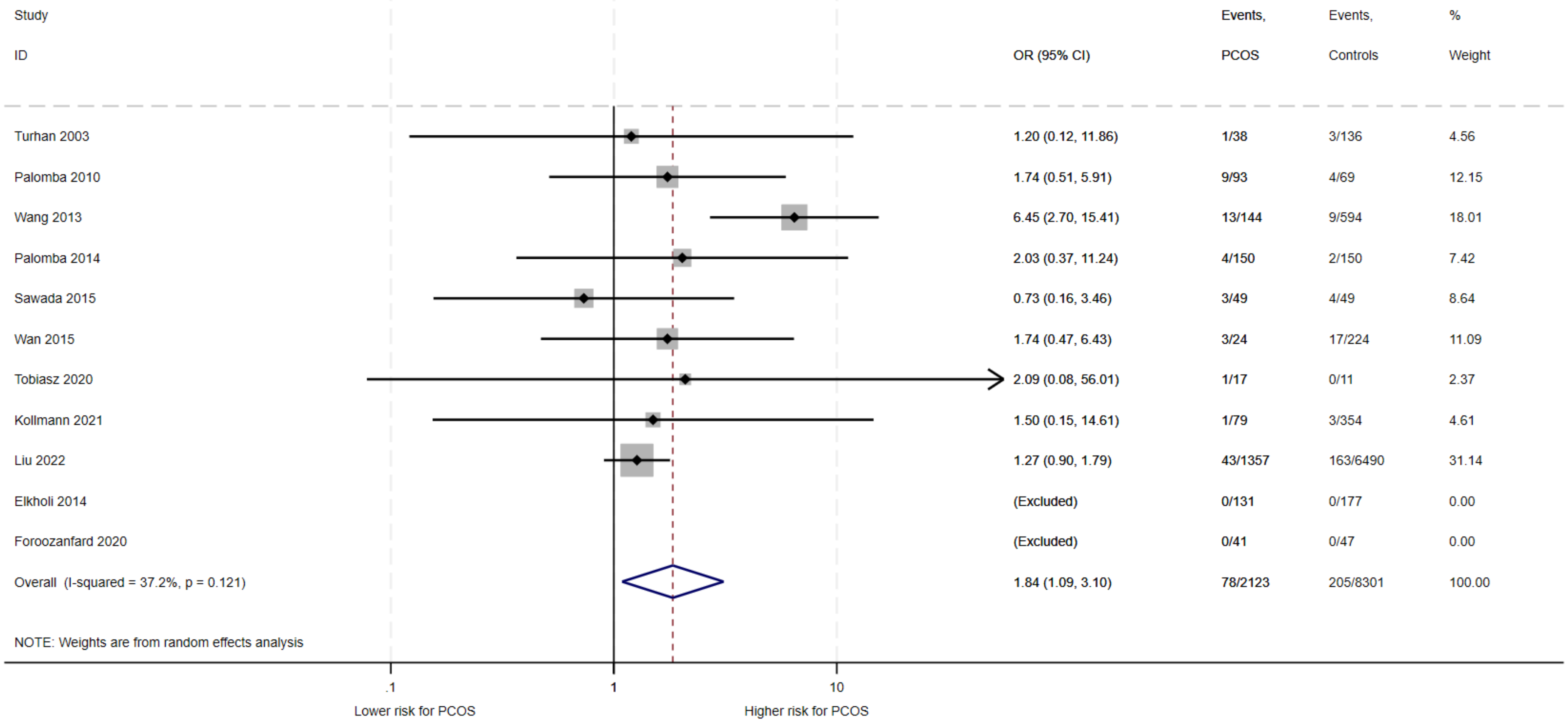

Supplementary Figure 3_b. Cumulative plot of association of polycystic ovary syndrome with fetal growth restriction

Convergence not achieved during tau^2 estimation

Supplementary Figure 3_c. Funnel plot assessing publication bias in studies on the association of polycystic ovary syndrome with fetal growth restriction

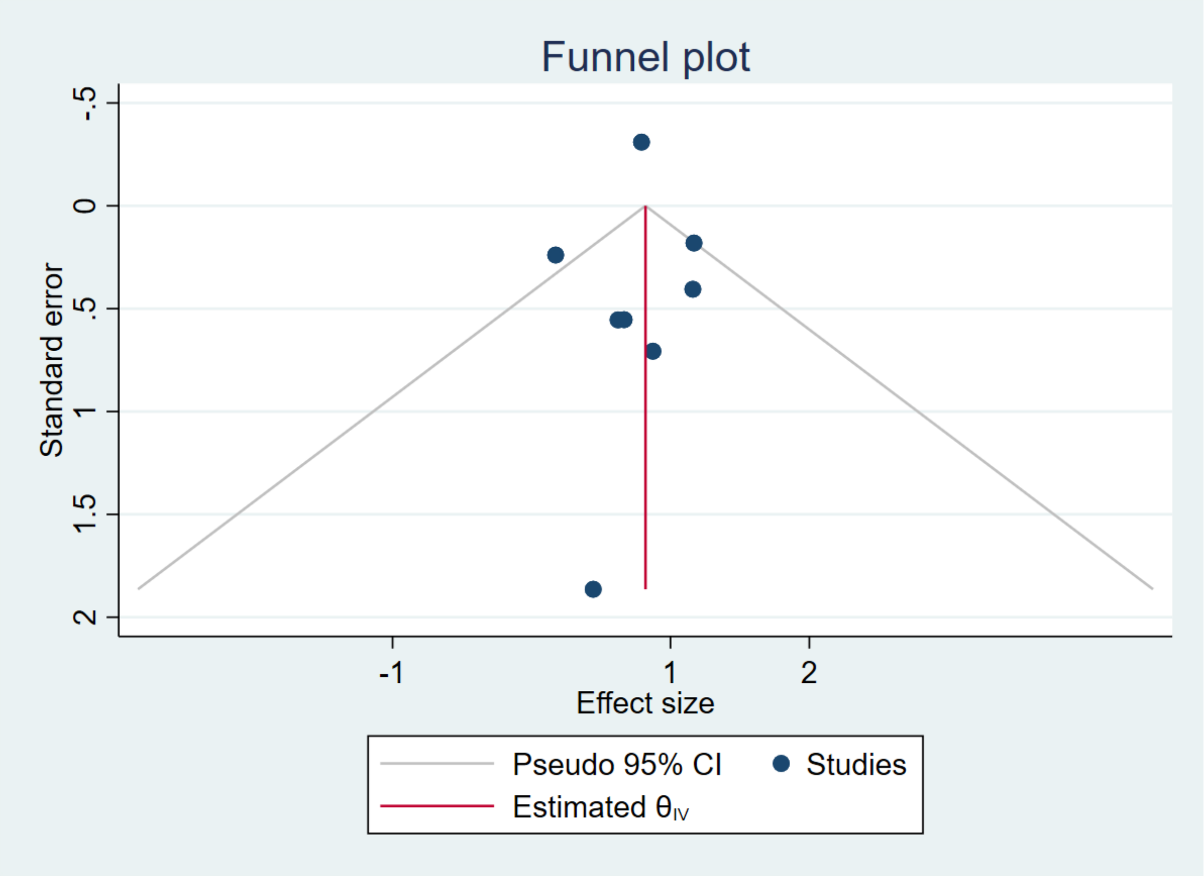

Source data are provided as a Source Data file.

Supplementary Figure 4_a. Forest Plot of association of polycystic ovary syndrome with low birth weight

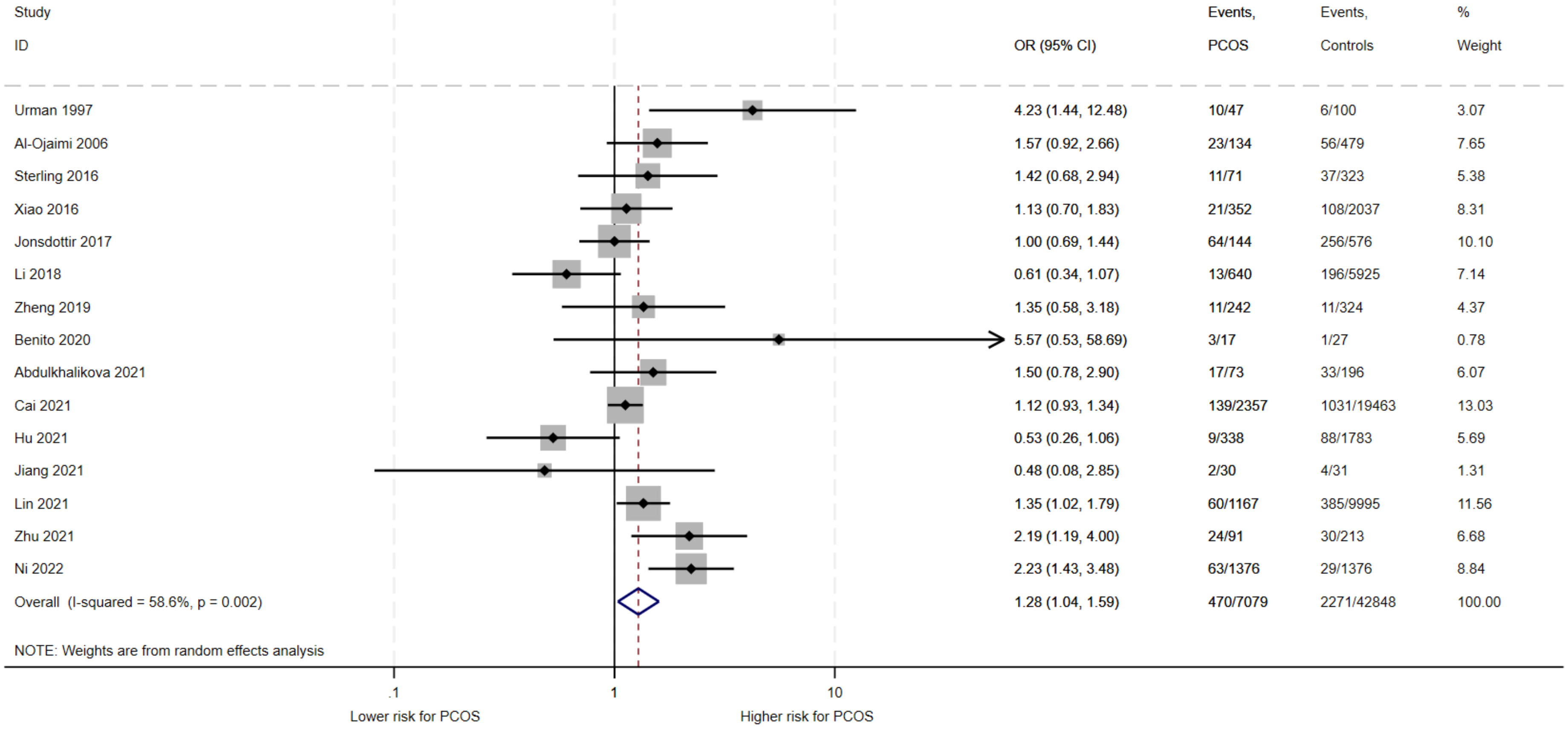

Supplementary Figure 4_b. Cumulative plot of association of polycystic ovary syndrome with low birth weight

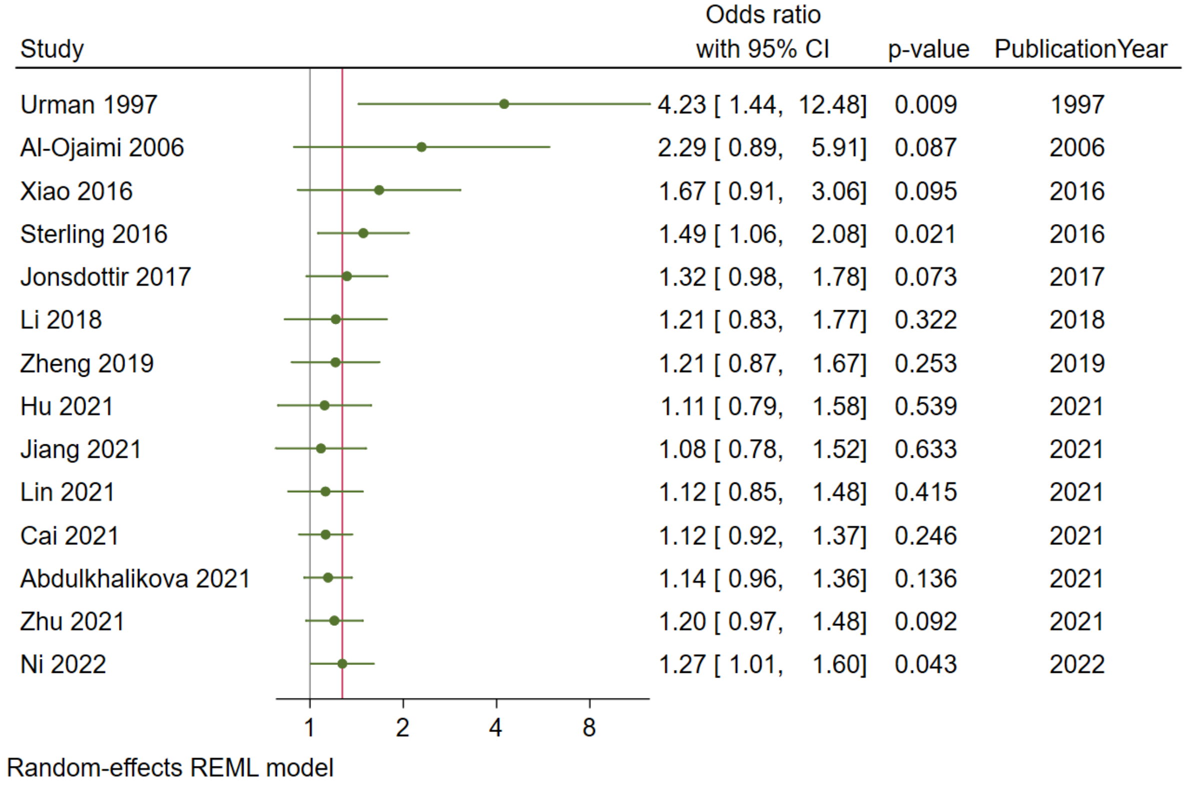

Supplementary Figure 4_c. Funnel plot assessing publication bias in studies on the association of polycystic ovary syndrome with low birth weight

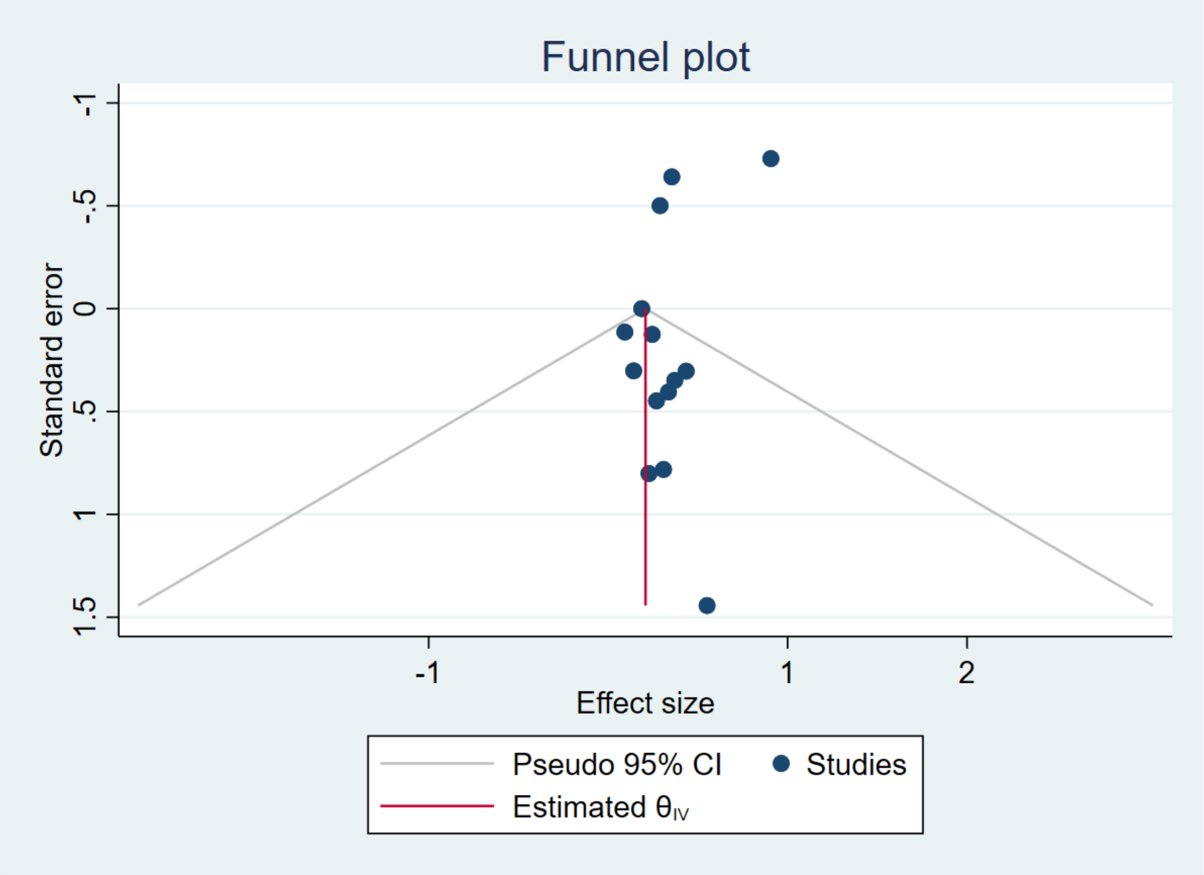

Source data are provided as a Source Data file.

Egger's test for small study effects on the outcome of low birth weight

Number of studies = 14 Root MSE = 1.622

Std_Eff	Coefficient	Std. err.	t	P>|t|	[95% conf. interval]	
slope	0.1286503	0.1840554	0.70	0.498	-0.272372	0.5296726
bias	0.3944567	0.8504814	0.46	0.651	-1.458583	2.247496

Test of H0: no small-study effects P = 0.651

Supplementary Figure 5_a. Forest Plot of association of polycystic ovary syndrome with small for gestational age

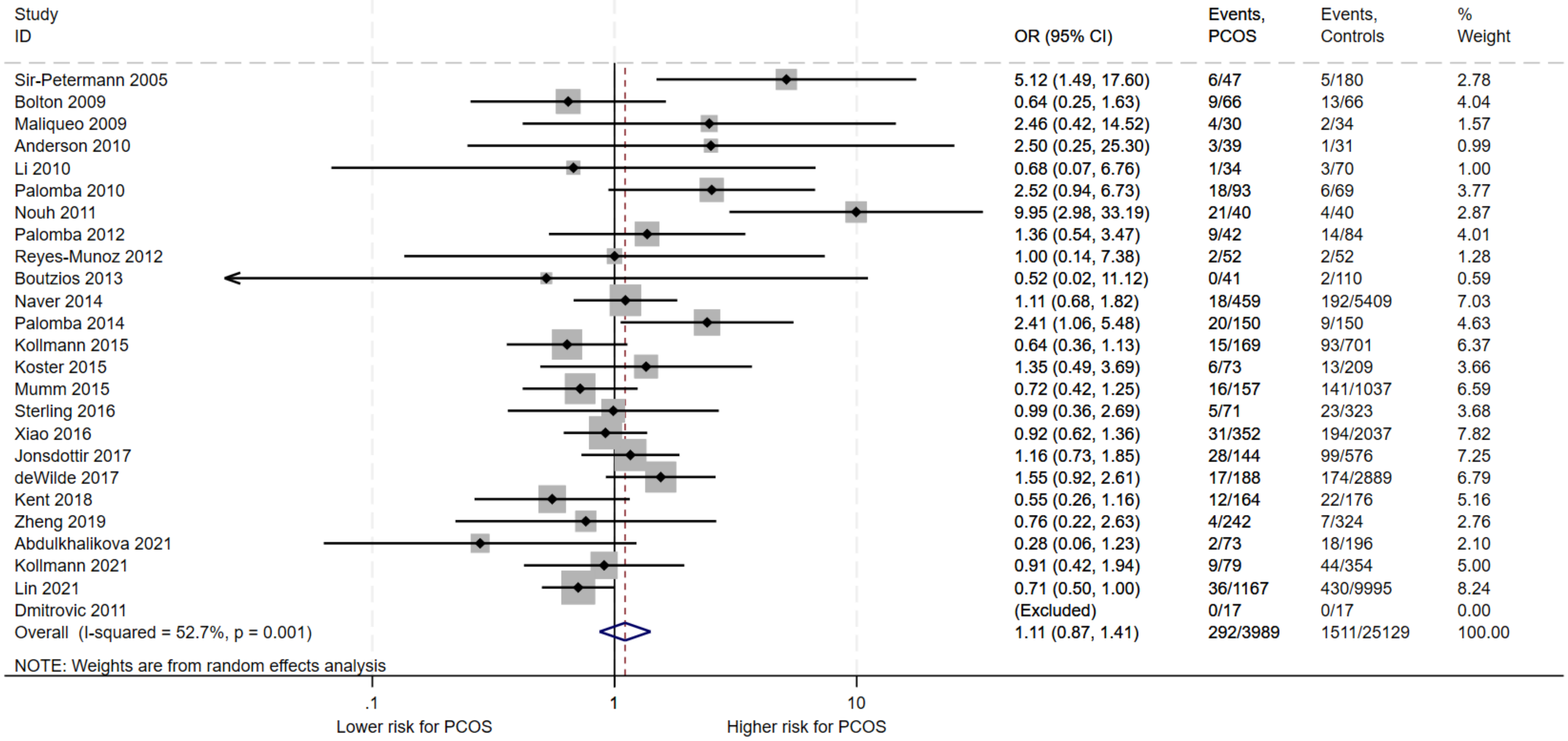

Supplementary Figure 5_b. Cumulative plot of association of polycystic ovary syndrome with small for gestational age

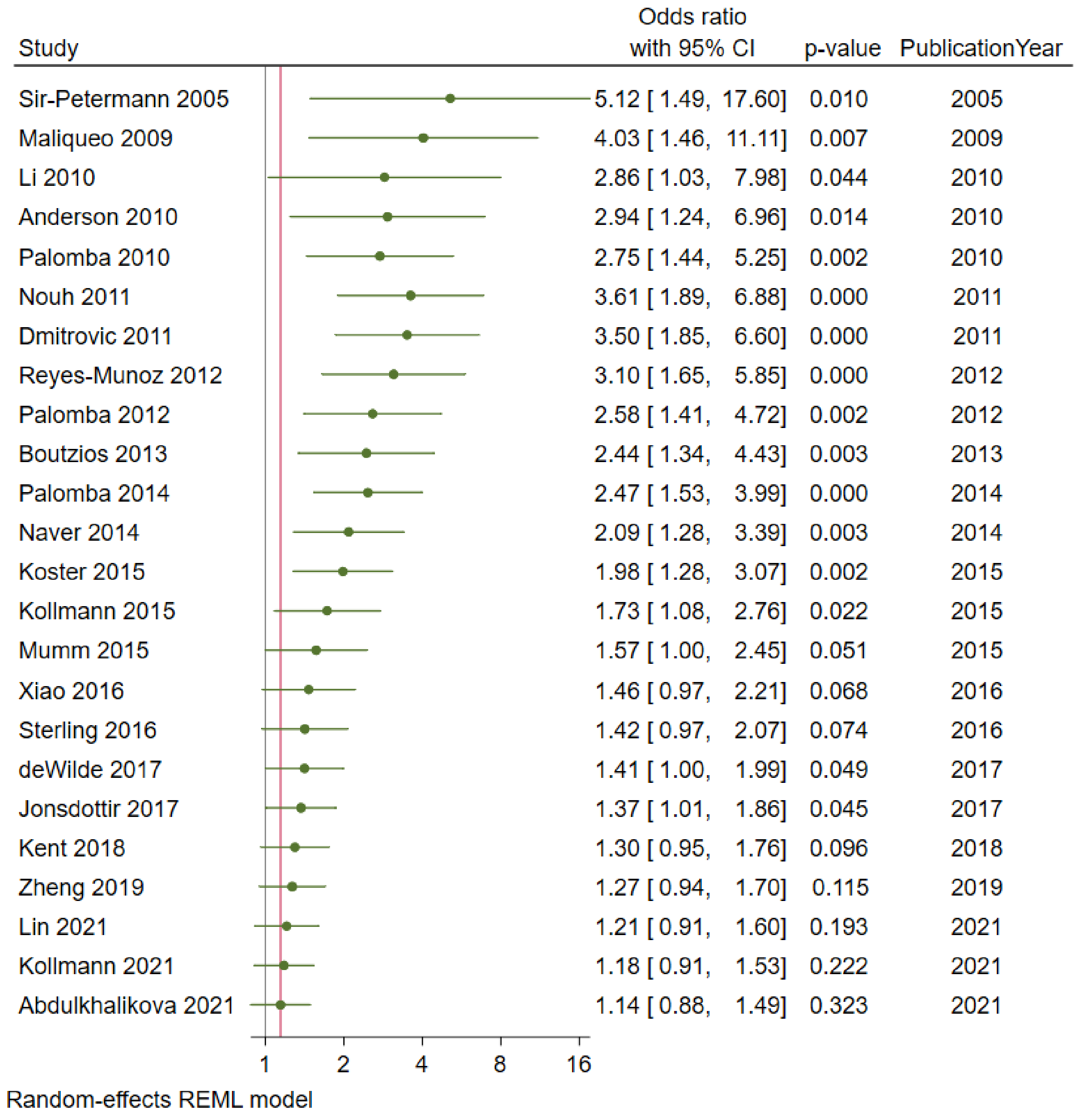

Supplementary Figure 5_c. Funnel plot assessing publication bias in studies on the association of polycystic ovary syndrome with small for gestational age

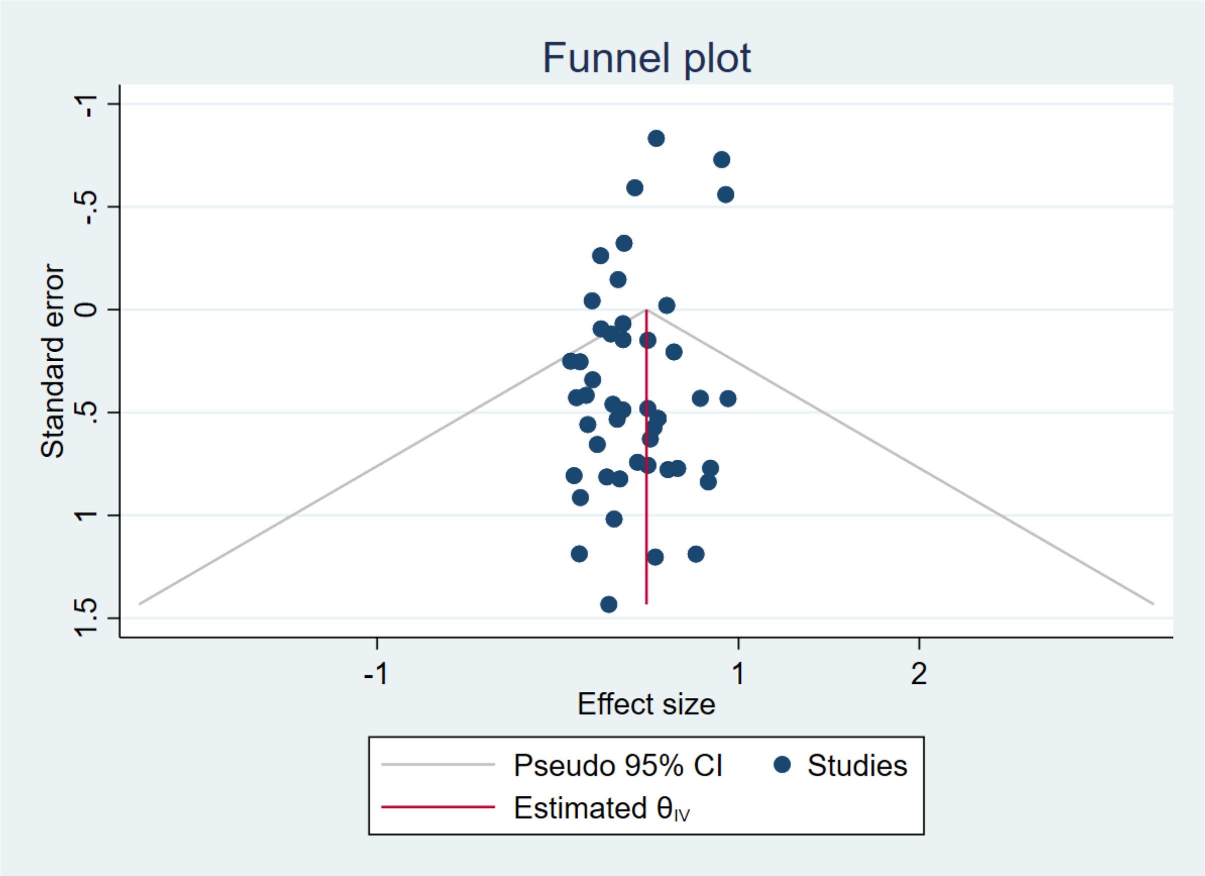

Source data are provided as a Source Data file.

Egger's test for small study effects on the outcome of small for gestational age

Number of studies = 23 Root MSE = 1.424

Std_Eff	Coefficient	Std. err.	t	P>|t|	[95% conf. interval]	
slope	-0.2883422	0.2181428	-1.32	0.200	-0.7419951	0.1653107
bias	0.9659631	0.6059285	1.59	0.126	-0.2941341	2.22606

Test of H0: no small-study effects P = 0.126

Supplementary Figure 6_a. Forest Plot of association of polycystic ovary syndrome with macrosomia

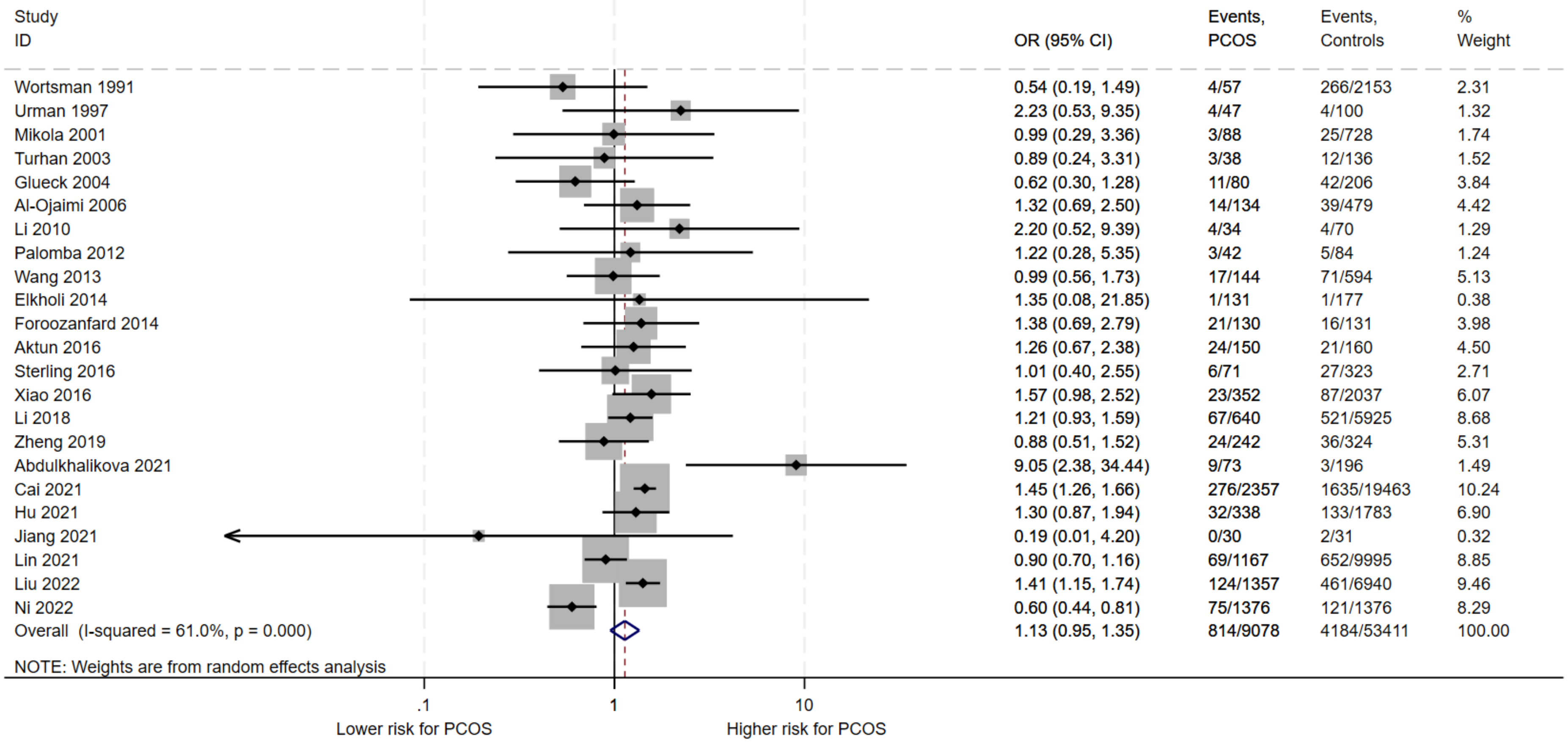

Supplementary Figure 6_b. Cumulative plot of association of polycystic ovary syndrome with macrosomia

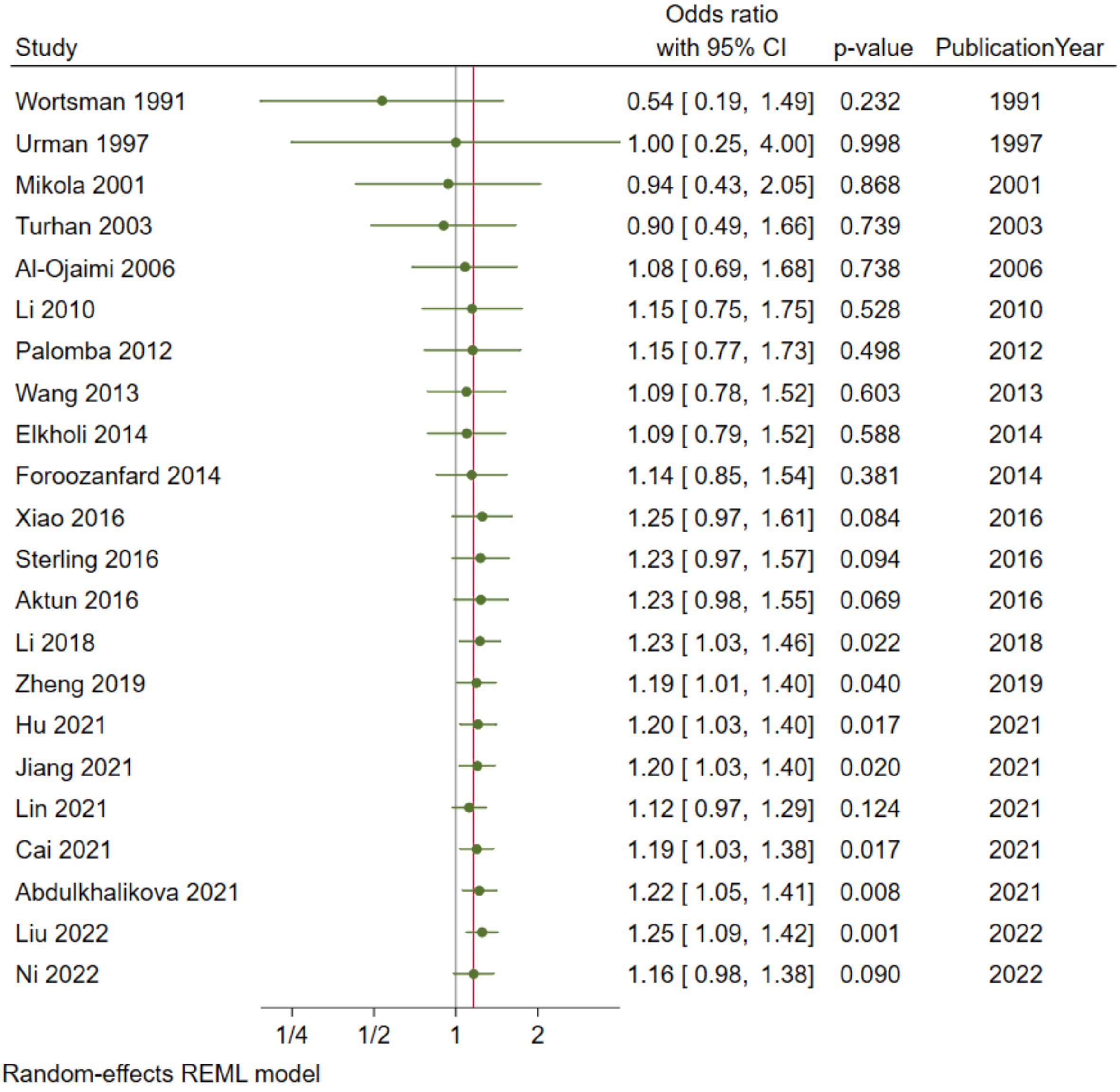

Supplementary Figure 6_c. Funnel plot assessing publication bias in studies on the association of polycystic ovary syndrome with macrosomia

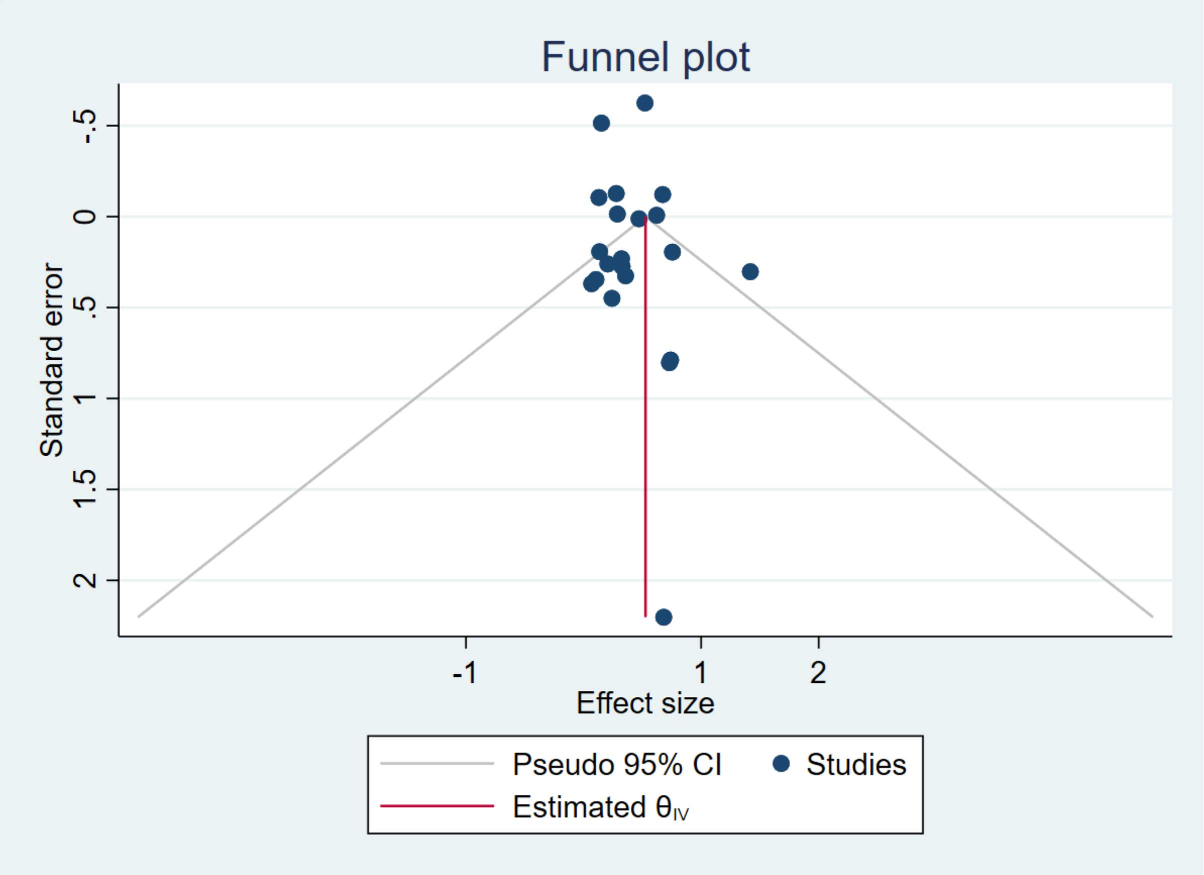

Source data are provided as a Source Data file.

Egger's test for small study effects on the outcome macrosomia

Number of studies = 22 Root MSE = 1.615

Std_Eff	Coefficient	Std. err.	t	P>|t|	[95% conf. interval]	
slope	0.2431784	0.1034699	2.35	0.029	0.0273441	0.4590128
bias	-0.2799458	0.5255781	-0.53	0.600	-1.376282	0.8163908

Test of H0: no small-study effects P = 0.600

Supplementary Figure 7_a. Forest Plot of association of polycystic ovary syndrome with large for gestational age

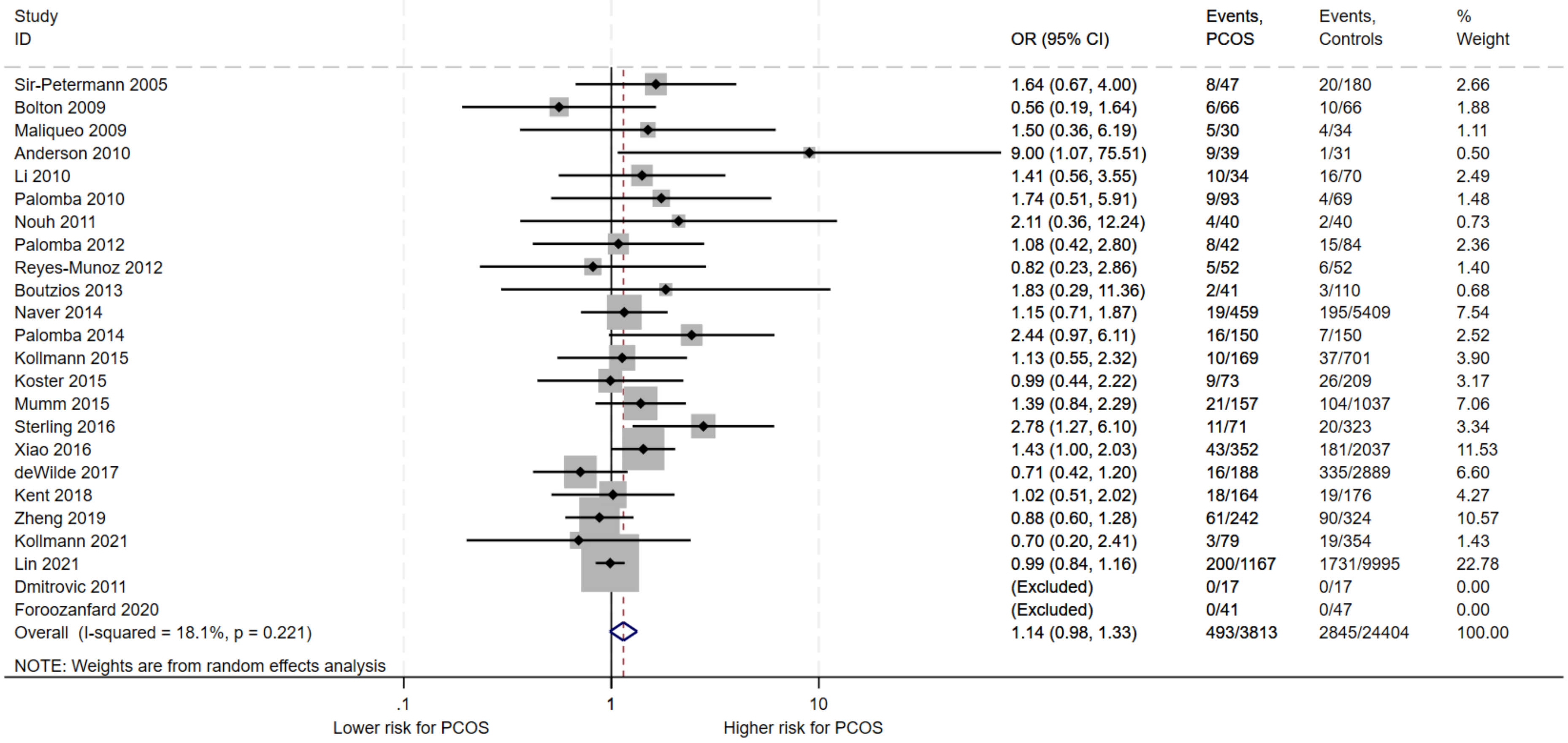

Supplementary Figure 7_b. Cumulative plot of association of polycystic ovary syndrome with large for gestational age

Study	Odds ratio with 95% CI	p-value	PublicationYear
Sir-Petermann 2005	1.64 [0.67, 4.00]	0.276	2005
Maliqueo 2009	1.60 [0.75, 3.40]	0.222	2009
Li 2010	1.52 [0.85, 2.73]	0.161	2010
Anderson 2010	1.72 [0.98, 3.03]	0.059	2010
Palomba 2010	1.73 [1.03, 2.88]	0.037	2010
Nouh 2011	1.75 [1.07, 2.86]	0.025	2011
Dmitrovic 2011	1.74 [1.07, 2.83]	0.026	2011
Reyes-Munoz 2012	1.57 [1.00, 2.48]	0.051	2012
Palomba 2012	1.47 [0.97, 2.21]	0.067	2012
Boutzios 2013	1.48 [0.99, 2.21]	0.053	2013
Palomba 2014	1.61 [1.11, 2.32]	0.011	2014
Naver 2014	1.42 [1.06, 1.90]	0.018	2014
Koster 2015	1.36 [1.04, 1.80]	0.027	2015
Kollmann 2015	1.33 [1.03, 1.72]	0.029	2015
Mumm 2015	1.34 [1.07, 1.69]	0.011	2015
Xiao 2016	1.37 [1.13, 1.66]	0.001	2016
Sterling 2016	1.42 [1.18, 1.71]	0.000	2016
deWilde 2017	1.32 [1.09, 1.61]	0.004	2017
Kent 2018	1.30 [1.08, 1.55]	0.005	2018
Zheng 2019	1.24 [1.03, 1.49]	0.023	2019
Foroozanfard 2020	1.24 [1.03, 1.49]	0.023	2020
Lin 2021	1.18 [1.00, 1.39]	0.046	2021
Kollmann 2021	1.17 [1.00, 1.37]	0.057	2021

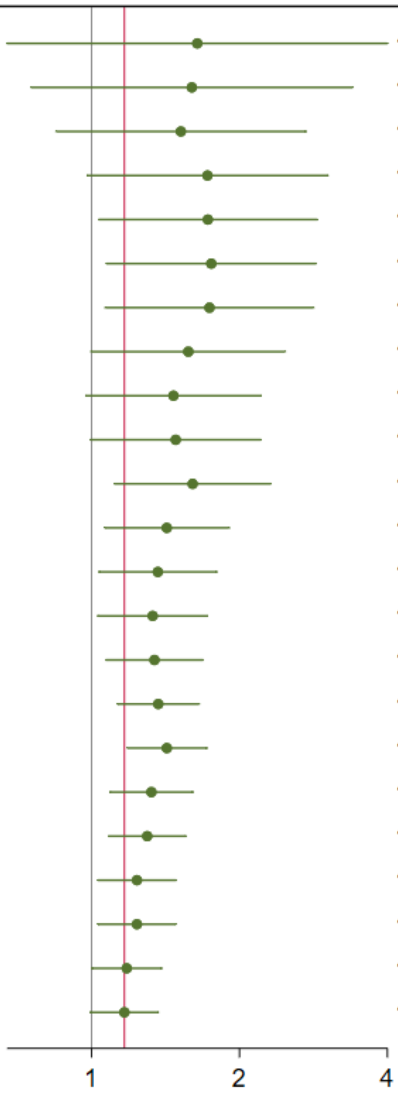

Random-effects REML model

Supplementary Figure 7_c. Funnel plot assessing publication bias in studies on the association of polycystic ovary syndrome with large for gestational age

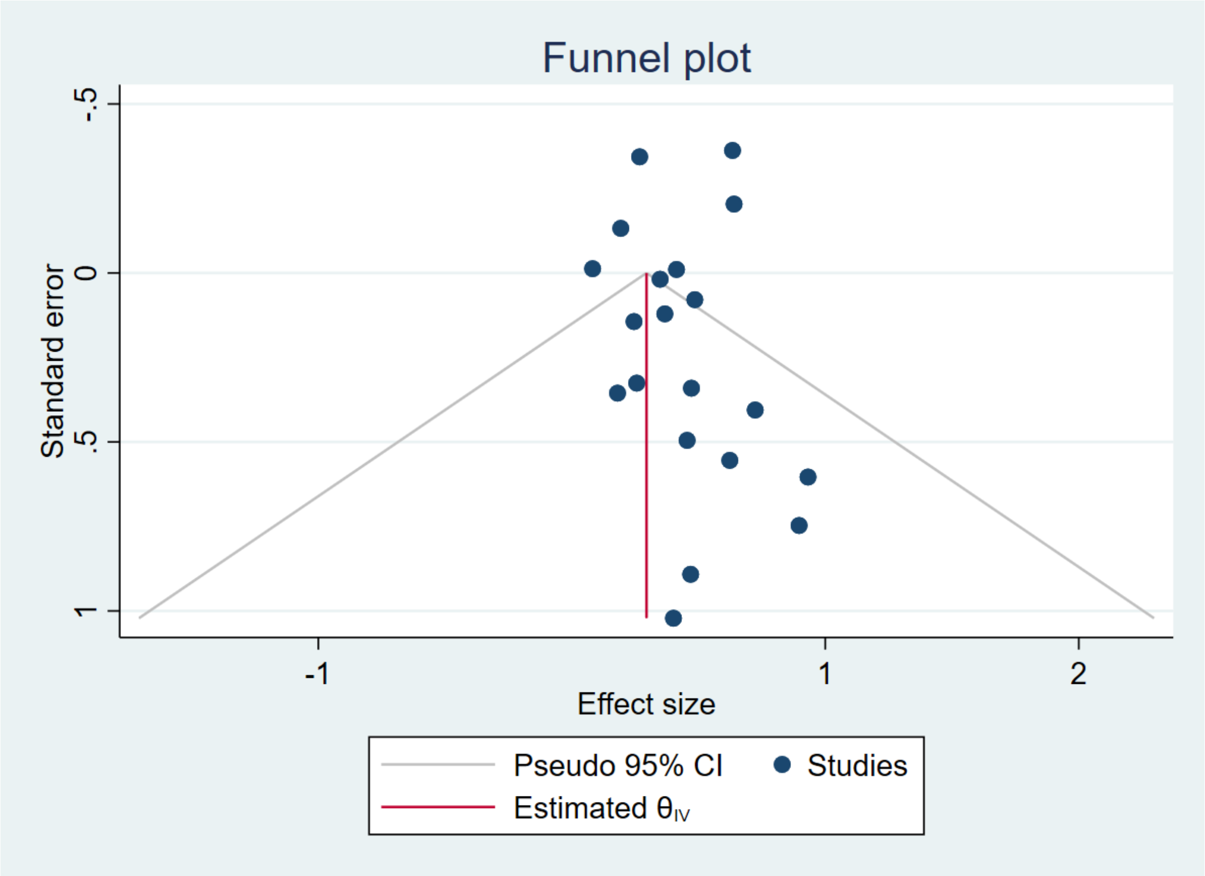

Source data are provided as a Source Data file.

Egger's test for small study effects on the outcome of large for gestational age

Number of studies = 21 Root MSE = 0.9627

Std_Eff	Coefficient	Std. err.	t	P>|t|	[95% conf. interval]	
slope	-0.0550578	0.088776	-0.62	0.543	-0.2415693	0.1314537
bias	0.6970052	0.349224	2.00	0.061	-0.0366871	1.430698

Test of H0: no small-study effects P = 0.061
